# Social Psychological Predictors of Belief in Fake News in the Run-Up to the 2019 Hungarian Elections: The Importance of Conspiracy Mentality Supports the Notion of Ideological Symmetry in Fake News Belief

**DOI:** 10.3389/fpsyg.2021.790848

**Published:** 2021-12-24

**Authors:** Zea Szebeni, Jan-Erik Lönnqvist, Inga Jasinskaja-Lahti

**Affiliations:** ^1^Swedish School of Social Sciences, University of Helsinki, Helsinki, Finland; ^2^Faculty of Social Sciences, University of Helsinki, Helsinki, Finland

**Keywords:** disinformation, conspiracy mentality, Hungary, fake news, personality, motivated reasoning, political orientation

## Abstract

Accessing information online is now easier than ever. However, also false information is circulated in increasing quantities. We sought to identify social psychological factors that could explain why some people are more susceptible to false information. Specifically, we investigated whether psychological predispositions (social dominance orientation, right-wing authoritarianism, system justification beliefs (SJB), openness, need for closure, conspiracy mentality), competencies (scientific and political knowledge, interest in politics) or motivated reasoning based on social identity (political orientation) could help explain who believes fake news. Hungarian participants (*N* = 295) judged political (anti- and pro-government) and non-political news. The Hungarian context—characterized by low trust in media, populist communication by the government and increasing polarization—should be fertile ground for the proliferation of fake news. The context in making this case particularly interesting is that the major political fault line in Hungary runs between pro- and anti-government supporter groups and not, for instance, between conservative and liberal ideology or partisanship. We found clear support for the motivational reasoning explanation as political orientation consistently predicted belief in both fake and real political news when their contents aligned with one’s political identity. The belief in pro-government news was also associated with higher SJB among pro-government supporters. Those interested in politics showed better capacity to distinguish real political news from the fake ones. Most importantly, the only psychological predisposition that consistently explained belief in all types of fake news was a conspiracy mentality. This supports the notion of ideological symmetry in fake news belief—where a conspiracy mentality can be found across the political spectrum, and it can make people susceptible to disinformation regardless of group-memberships and other individual differences.

## Introduction

According to a 2018 Eurobarometer, 83% of EU citizens feel that fake news represents a danger to democracy (European Commission, 2018). The spread of disinformation has been argued also by researchers to have the potential to undermine science and society, and cause further political polarization (e.g., Tucker et al., 2018). Fake news, or disinformation can be defined as “misinformation coupled with a clear intent to cause harm or purposefully deceive others” (van der Linden, 2017, p. 5). Recent comparative research has identified the following macro-level conditions as increasing the overall vulnerability to disinformation: polarization, populist communication, low trust in the news, weak public service media and media regulation, fragmented audiences, a large advertisement market and high social media use (Humprecht et al., 2020). Yet, to date little research has been run in environments in which these societal characteristics are particularly extreme, like contemporary Hungary. In addition to macro-level societal factors, researchers have investigated the fabric of the fake news and its production by focusing for example on whether some features of fake news (e.g., source, internal consistency) make disinformation more compelling (e.g., Schaewitz et al., 2020). Research has also identified several individual difference variables related to cognitive processing which can affect the accuracy ratings of disinformation (Pennycook and Rand, 2020; Scherer et al., 2021). However, we still know little about the possible social psychological factors associated with susceptibility to disinformation.

The aim of the current study is to identify social psychological predictors of the misclassification of fake and real news. The novelty of our research lies, first, in its holistic nature, we explore not only fake, but real news, political and non-political, to see how the content and narratives of the news pieces is evaluated by individuals with certain psychological predispositions, competences, and political identities. Second, we investigate fake and real news belief in Hungary, a context in which fake news are highly prevalent, and political polarization is extremely high (Krekó and Enyedi, 2018). Comparing our results with results obtained in less extreme contexts could be highly illuminating with regards to the generalizability of the existing finding obtained mostly in a democratic Western context (e.g., Pennycook and Rand, 2018). A third novelty lies in the fact that Hungary is not polarized according to partisanship lines or to political ideology, but instead the main political cleavage runs between pro- and anti-government supporter groups. This is important, as it allows us to rule out the possibly confounding effects of political ideology—i.e., political conservatism has been associated with several of the psychological predispositions that we investigate (Jost et al., 2003), as well as with engagement with disinformation (Guess et al., 2020).

Our results could help create context-specific procedures to guard against the proliferation of false information among individuals particularly prone to misclassifying fake and real news. Moreover, by examining competencies, political identities as well as psychological predispositions, our results can contribute to the design of possible future interventions.

### Deficit and Motivated Reasoning

Why do people believe disinformation? Currently there are several explanations as to why people might be susceptible to disinformation in the online sphere. The first set of explanations involve some form of deficit, such as lack of political or scientific knowledge or lack of online skills, that explains why the person is unable to discern false news from real news. It seems intuitive to assume that people, who know less about a certain topic are more susceptible to disinformation in that area. However, results regarding this connection are mixed: lower health literacy has been connected to being more susceptible to disinformation (Scherer et al., 2021), but people who know less about politics may not be more likely to fall for disinformation, as fake news consumption is not lower among politically knowledgeable individuals (Guess et al., 2018). While more research is needed to contextualize and better understand the connection between age and online information processing, during the 2016 presidential campaign in the US, people above 65 shared false information seven times more, than did younger groups. This might be because of the lack of digital media literacy, which would be needed to determine the trustworthiness of the given information (Guess et al., 2019).

Another set of explanations underlines motivated reasoning, that is, that people are motivated to believe what they want to believe and what dovetails with their worldviews and prior knowledge. That is, people tend to arrive at the conclusions that they want to arrive at Kunda (1990), thus accepting fake news that line up with their ideology (Nyhan and Reifler, 2010). This explanation is consistent with research suggesting that liberals and conservatives show similar levels of partisan bias in judging fake news (Ditto et al., 2019). Confirmation bias refers to the bias people have in seeking and interpreting information that aligns with their existing identities, expectations, and attitudes (Nickerson, 1998). Partisan motivated reasoning refers to higher acceptance of political information that is consistent with one’s ideology or partisanship, regardless of the accuracy of that information (e.g., Nyhan and Reifler, 2010; Van Bavel and Pereira, 2018). Supporting the importance of existing attitudes, research shows that corrections made to reduce misperceptions among targeted ideological groups often fail (Nyhan and Reifler, 2010). Similarly, flagging fake news as fake does increase cognitive activity, but does not stop social media users from believing headlines that align with their political views (Moravec et al., 2018). For the purpose of exploring the motivated reasoning account, we distinguished between the political narratives underlying different types of (fake) news.

### Psychological Predispositions

In addition to deficit and motivated reasoning approaches, the third set of explanations for why some people believe fake news focuses on underlying psychological predispositions. The notion of ideological asymmetry in disinformation belief, according to which conservatives are more likely than liberals to believe fake news (e.g., Guess et al., 2018; Hjorth and Adler-Nissen, 2019), has its roots in this type of explanation. However, exactly, which psychological predispositions could underlie such asymmetry has not been established. We next provide a rationale to expect some particular psychological predispositions to be crucial in understanding individual differences in the consumption and evaluation of disinformation.

Conservatism is closely associated with social dominance orientation (SDO) and right-wing authoritarianism (RWA) (Duriez and Van Hiel, 2002), and this could help explain the above mentioned differences between conservatives and liberals in engagement with fake news (e.g., Haidt, 2013; Jost, 2017). SDO is a preference for group-based social hierarchy and support for inequality between social groups (Sidanius and Pratto, 2001). Higher SDO has been associated with greater in-group favoritism and in-group bias (Sidanius et al., 1994). RWA is, in turn, defined by the traits of authoritarian submission, endorsement of traditional social norms and conventions (Altemeyer, 1981). Authoritarians are motivated both to preserve traditions and ingroup norms (Duriez and Van Hiel, 2002) and to punish groups that are perceived to threaten the norms and rules of the ingroup (e.g., Hadarics and Kende, 2018).

Conservatism, RWA and SDO have all been shown to serve system-justifying functions (Jost and Hunyady, 2005). SJB include the inclination to rationalize and justify the current *status quo* and to perceive the system as reasonable, beneficial and fair (Jost and Banaji, 1994). In this line of thinking, threats to the current political arrangement are thought to also threaten psychological needs, causing people to defend the system even at their own expense (Jost et al., 2004). System justifying motives can also cause people to actively avoid threatening information (Shepherd and Kay, 2012), and conservatives are generally more inclined to endorse system-justifying attitudes than liberals (Jost et al., 2008). Furthermore, both the need for cognitive closure and openness to experience have been found to be dispositional antecedents of SJB (Jost and Hunyady, 2005). We intend to investigate whether RWA, SDO, or system justifying beliefs— psychological predispositions all of which underlie conservatism—could help explain susceptibility to fake news.

Personality traits can affect people’s judgment in a variety of situations (e.g., Byrne et al., 2015), implying that also other psychological predispositions than those associated with conservatism could be relevant for the ability to accurately judge news headlines. Those with a pronounced need for cognitive closure have been described as striving to eliminate uncertainty (Webster and Kruglanski, 1997), form judgments swiftly on a given issue (Kruglanski et al., 1991) and show less information-seeking behavior (e.g., Klein and Webster, 2000). The need for cognitive closure has also been associated with belief in conspiracy theories (Marchlewska et al., 2018). Openness, in contrast, refers to the urge for experiences as well as the tendency toward cognitive exploration (Kaufman et al., 2016), and is associated with more effortful information seeking, while those low in openness have been found to prefer the confirmation of familiar information (Heinström, 2003). Sindermann et al. (2020) in a recent review suggest that openness should act as a buffer against fake news belief, and some research seems to support this, as higher openness has been found to be associated with being better at discerning fake from real news (Heinström, 2003; Calvillo et al., 2021) and lower susceptibility to misinformation (Doughty et al., 2017). However, Wolverton and Stevens (2019) found the exact opposite, that participants who scored low on openness were better at identifying false information than those who scored high, while Sindermann et al. (2021) found no major role of openness explaining any tendencies of fake news discernment.

Another psychological predisposition that might explain the susceptibility to disinformation is belief in conspiracy theories. The concept of a conspiracy mindset (also called a conspiracy mentality or conspiracy thinking; Imhoff and Bruder, 2014) has been put forward as a relatively stable personality characteristic describing individual differences in the extent to which people believe in conspiracies or conspiracy theories (e.g., Moscovici, 1987; Imhoff and Bruder, 2014). Conspiracy theories, explain the reasons behind important political and social events by means of secret plots by two or more powerful actors (Aaronovitch, 2014; Dentith and Orr, 2018). Any group can be accused of conspiring, as long as they are perceived as powerful and malicious (Douglas et al., 2019).

While disinformation can contain conspiratorial narratives, conspiracy theories differ from disinformation in that they are speculative, complex and resistant to falsification, and that belief in them serves existential, epistemic and social motives (Douglas et al., 2017). The epistemic motivation stems from the need to reduce uncertainty, and to build a stable, accurate and consistent understanding of the world. Thus such a personality disposition is stronger among people who seek patterns in their environment (Bruder et al., 2013) or have higher need for cognitive closure (Marchlewska et al., 2018). Existential motives mean the need to feel safe and secure in the environment, thus they relate to lack of sociopolitical control (Bruder et al., 2013). Social motivations include the need to maintain a positive image of the self and the group we belong to, through allocating blame on “the others,” which can help to uphold a competent image of the ingroup (Cichocka et al., 2016). Although both conspiracy theories and disinformation include misperception or ignorance of reality, belief in disinformation is believing something specific that is factually false, and this belief may or may not act as a building block for a conspiratorial worldview. This means that belief in disinformation should, when not part of an altogether conspiratorial worldview, be less resistant to being corrected (Scheufele and Krause, 2019). Despite these distinctions, disinformation and conspiratorial worldviews tend to be tightly connected in the real world, as conspiracy theorists are one of the main producers of disinformation (Tucker et al., 2018).

### Fake News in Hungary

Hungary has seen the continuous decline of press freedom since 2010, and was ranked at 92nd in the 2021 World Press Freedom Index by Reporters without Borders (2021). The government led by Viktor Orbán has been in power since 2010 with a supermajority in parliament, which allowed the complete transformation of the country’s media sphere.

In 2010 a law-package that severely curbed media freedom in the country was passed. It granted the Media Council regulatory power over all forms of media, and its members were appointed for 9 years, by the Parliament and the Prime Minister. In 2019, only the nominees of Fidesz were elected as new members (Haraszti, 2011). The government not only influences the public media, but private media, through government-friendly private actors who own private media outlets. In 2018, KESMA (Central European Press and Media Foundation) was created, to which more than 470 Hungarian media outlets were transferred from said private individuals. Besides KESMA, the Hungarian media landscape is generally distorted by state advertising and censorship, which help create a government-friendly media empire that constitutes a potent tool of political favoritism (Bátorfy and Urbán, 2020). The situation of independent journalists has also worsened; they are often banned from certain events, and government politicians do not give interviews to independent media outlets.

Through the government-friendly private and public media Fidesz, like no other political party, has been able to communicate their political messages in the online and offline sphere, through “public service” messages, financed by the government, party messages are communicated on several media outlets. While direct political control over public media is not widespread, it is commonly known that indirect influence is exerted over these mediums, resulting in the spread of pro-government messages (European Commission [EC], and Directorate-General for Justice and Consumers, 2020). These also involve disinformation, for example the employees of the taxpayer-funded national MTVA network admittedly fabricated false stories on immigration, supposedly on the order of the government (Nolan and Walker, 2018). Government-friendly media has spread pro-Kremlin conspiracies (Győri and Krekó, 2017) and political and non-political fake news are prevalent also in state-funded and government-friendly private media (Krekó and Enyedi, 2018).

At the time of the present research, Hungarian politics was increasingly divided into two camps: the government and the opposition. In the opposition, parties across the political and ideological spectrum were increasingly working together, coordinating candidates in the 2019 local elections (Agence France-Presse, 2019). The united opposition includes an ideologically extremely diverse set of parties. For instance, the far-right party Jobbik, which on several issues positions itself as even further to the right than Fidesz (Goldstein, 2021), is united with parties that position themselves as left-wing [Hungarian socialist party (MSZP), democratic coalition (DK), dialogue for Hungary (PM)]. Also part of the mix is a young, liberal party (Momentum), and a Hungary’s green party (LMP). Many of these parties have nothing in common in terms of ideology or policy, but they are held together by an anti-Orbán, anti-corruption and pro-democracy sentiment. Humprecht et al. (2020) describe such “two-party” and winner-takes-all systems as fertile ground for political polarization as well as affective polarization between the two opposing camps. In such an environment it can be increasingly difficult to judge the accuracy of information (Craft et al., 2017). Furthermore disinformation can be especially effective in Hungary, as it is marked by one of the lowest levels of media trust in the EU, with 52% of the people claiming to come across false information almost every day (European Commission, 2018).

### Purpose of the Present Research

In sum, the present study aims to clarify which social psychological factors are associated with believing fake and real news in a Hungarian context. Our research advances the previous line of research in several ways: first, research on disinformation often concentrates on one type of disinformation, such as a specific health topic (e.g., vaccines), or political disinformation. For a broader perspective, we included political (dis)information with different narratives as well as non-political disinformation. Second, unlike previous research that included some single personality factors or competences in their models, we tested the simultaneous associations between several understudied psychological predispositions (such as SDO, RWA, SJB, openness, need for cognitive closure and a conspiracy mentality), competencies (political and scientific knowledge and political interest) and political orientation based on shared socio-political identification, which all potentially underlie fake news susceptibility. Whether not only personality but also acquired competencies and political identities matter is especially important from the perspective of developing interventions—could for instance educational interventions that provide more knowledge or aim at groups’ recategorization help? Third, we explored disinformation in the highly politically polarized context of Hungary, which, like almost all other non-Western or non-democratic contexts, is under-researched. Moreover, the current political divide in Hungary is not based on ideology but on stance toward the current government. This last point is especially important; when the political divide is ideological (e.g., conservatives vs. liberals), it may in part be built on the same psychological predispositions that are then used to explain belief in fake news. That is, if, as in the US, conservatives are more prone to believe in fake news (e.g., Guess et al., 2020) this may be because some underlying psychological dispositions (e.g., RWA, SDO) have made them both more ideologically conservative and more susceptible to fake news. However, in the Hungarian context, those on the anti-government side can be anywhere on the ideological map, eliminating spurious associations between ideological bent and susceptibility to fake news.

Our main general research questions are thus: *Who is susceptible to disinformation? Are there differences in the social psychological characteristics of the people who believe the different types of disinformation? What are the most important social psychological predictors of disinformation susceptibility?*

## Materials and Methods

### Participants and Procedure

Participants completed an online questionnaire in 2019 between April 8th and May 20th. In this time window, we sought to recruit as many participants as possible. We decided to end the data collection 1 week prior to the 2019 European Parliament elections, to keep the election and the election results from interfering with the results. Participants were a convenience sample recruited through social media. Master’s students posted the questionnaire on Facebook and shared it in their social circle. It was also posted in political discussion groups. The 35-min questionnaire was described as being part of a study on information processing, in which we ask about participants’ perception of news, politics and societal issues. Altogether 702 Hungarian participants took part. Participants were not monetarily compensated. They could quit at any time and were allowed to skip questions (some of the questions were somewhat sensitive, given the political context), but we excluded those participants who failed to complete at least 99% of the questionnaire. Those excluded had a mean progress of 24, and 80% did not reach 40% completion. The final sample included 295 participants (mean age = 36.41), 115 males and 169 females (4 others, 8 missing). Just 5.1% completed primary school, 49.4% secondary school, and 42.5% graduated from higher education (3% missing). The research was conducted with the approval of the Ethics Committee of Eötvös Loránd University, Budapest, Hungary.

### Measures

#### Belief in News

We presented participants real news headlines, taken from Hungarian news websites in the months prior to data collection. They were shown in ‘Facebook’ format, with a headline, an associated photograph, and a byline below the photo. Sources were removed in order to omit their probable influence on the judgment of the information. For every piece of news, participants responded to the question “To what extent do you think this news is true?” on a scale of 1–7 (1 = *I am very sure this news is not true*, 4 = *the news is as likely to be true as it is likely to be false* and 7 = *I am very sure that this news is true*). We utilized a scale instead of dichotomous judgment, because they measure belief in a more subtle way than dichotomous judgments, thus it also gave participants the chance to claim that they are undecided on the given item. Furthermore such scales have been utilized in previous studies as well (e.g., Pennycook and Rand, 2018; Faragó et al., 2019; Calvillo et al., 2021).

In all, 18 pieces of news (fake: three pro-government, three anti-government, three non-political; real: three pro-government, three anti-government, three non-political) were presented in random order. The news items can be found in the Supplementary Materials and examples for all types of news are presented in Table 1. Our index of belief in a particular type of news was computed as average belief across the three news items in that category. Cronbach’s α for belief in anti-government fake news was 0.65, for anti-government real news 0.40, non-political fake news 0.37, non-political real news 0.45, pro-government fake news 0.71 and pro-government real news 0.34. Spearman–Brown Coefficient for belief in anti-government fake news was 0.692, for anti-government real news 0.389, non-political fake news 0.464, non-political real news 0.509, pro-government fake news 0.621, and pro-government real news 0.251.

**TABLE 1:** Examples of the different news types included in the study, including the headline and byline.

	Fake	Real
Political, pro-government narrative	Peter Juhász regularly abused drugs in front of his children As we earlier reported, shocking details came to light from the files of the court case of Peter Juhász: the partner of the president of Együtt said, that he gave her tranquilizers, after he knelt on her and hit her	GDP grew with 4.8% last year Hungary’s gross domestic product grew with 4.8% in last year’s last quarter, when accounting for seasonal and calendar effects. According to raw data, it grew by 5% compared to the same period of the year before – reported the KSH Thursday morning.
Political, anti-government narrative	A whole floor is reserved for Viktor Orbán, at a secret private clinic in Graz. Our source was not willing to tell us anything about the illness of the prime minister, but they said, that the most modern equipment and neurologists who studied at the best places are at the disposal of the important guest.	Here is the letter of Orban, asking for George Soros’ help. Most people know, that Viktor Orban studied at Oxford before the end of communism in Hungary. 30 years passed, and many things changed in the mind of the prime minister.
Non-political	Herb man from Bükk: everything is healable! To prevent cancer: soda bicarbonate, for childless: celery and quail egg yolk!	Pedestrians die because of healthy lifestyle, mobile phones and big cars. Since 1990, last year was the highest in pedestrian deaths caused by accidents

*The news in the questionnaire appeared with a picture in the original Hungarian format, translation is made by the authors.*

In selection of news, we followed the method of similar studies (e.g.: Pennycook and Rand, 2018; Calvillo et al., 2021; Scherer et al., 2021). The political news had to include a politician, a party, the government, or a policy, while the non-political news had to be clearly free of direct political, or politicized narratives in the given context (e.g., vaccines). The co-authors had to agree on whether each news item was true or false.

#### Political Orientation

We assessed political orientation by asking participants’ satisfaction with the government. giving us the pro- and anti-government camps. Hungary’s political arena was at the time increasingly divided by the support of the government, not by a more traditional conservative/liberal or left/right divide. Government-opposition dynamics have also more generally been shown to be the main drivers of voting behavior as opposed to conservative/liberal or left/right policy positions (Hix and Noury, 2016). Also importantly, political terms such as left and right do not necessarily have a coherent meaning in post-communist countries (Piurko et al., 2011).

Participants indicated their satisfaction with the government by responding to two questions: “Thinking about the Hungarian government, how satisfied are you with the way it is doing its job?” (on a scale of 0–10, 0 *extremely dissatisfied*, 10 = *extremely satisfied*), “On the whole, how satisfied are you with the way democracy works in Hungary?” (on a scale of 0–10, 0 = *extremely dissatisfied*, 10 = *extremely satisfied*). The average score of these two items constituted our measure of general satisfaction with the government. The questions were taken from the European Social Survey. Spearman–Brown Coefficient was 0.923.

#### Political Interest

We measured political interest with three questions: “How interested would you say you are in politics – are you” (on a scale of 1–5, 1 = *not interested at all* and 5 = *very interested*), based on Danckert et al. (2017), “How aware are you of current politics?” (on a scale of 1–5, 1 = *I don’t follow current politics* and 5 = *I am very much aware of current politics*) and “Compared to the general population how knowledgeable are you on current politics?” (on a scale of 1–5, 1 = *much less*, 5 = *much more*). The average score of these three items constituted our measure of general political interest. Cronbach’s α was 0.87, Spearman–Brown Coefficient was 0.887.

#### Political Knowledge

To measure political knowledge participants completed a test that consisted of 10 multiple choice questions—with 4 possible answers—related to current political and relevant historical events. For instance, “Who is the current Speaker of the National Assembly? A. Kövér László, B. Semjén Zsolt, C. Orbán Viktor, D. Áder János.” Participants had 15 s per question to respond. The total scores were calculated by adding up the right answers (one point per correct answer).

#### Scientific Knowledge

Scientific Knowledge was measured by six questions, two of which were multiple choice (two and three options), and four of which were true/false statements, such as “Antibiotics kill viruses as well as bacteria.” The questions are used by the National Science Foundation of the United States (National Science Board, 2010). The total scores were calculated by adding up the right answers (one point per correct answer).

#### Conspiracy Mentality

Conspiracy mentality was measured using the Hungarian version (translated by Orosz et al., 2016) of the 5-item Conspiracy Mentality Questionnaire (Bruder et al., 2013). Participants rated their agreement with statements, such as “I think that many very important things happen in the world, which the public is never informed about,” using a scale of 1–7 (1 = *not true at all*, 7 = *completely true*). The average score of these five items was calculated as a score of conspiracy mentality. Cronbach’s α was 0.79, Spearman–Brown Coefficient was 0.786.

#### Openness

Openness was assessed by the 10 openness items of the Hungarian version (Szirmak, 2009) of the 60-item HEXACO Personality Inventory (Ashton and Lee, 2009). Participants rated their agreement with the statements, such as “I would be quite bored by a visit to an art gallery,” using a scale of 1–7 (1 = *strongly disagree*, 7 = *strongly agree*). The score was calculated as the average value of the scale items. Cronbach’s α was 0.66.

#### Need for Closure

Need for closure was measured by the short, 15-item Hungarian version (Csanádi et al., 2009) of the Need for Closure Scale (Roets and Van Hiel, 2011). Participants rated their agreement with statements, such as “I don’t like situations that are uncertain,” using a scale of 1–6 (1 = *strongly disagree*, 6 = *strongly agree*). The score was calculated as the average value of the scale items. Cronbach’s α was 0.84.

#### System Justification Beliefs

System justification was measured by the Hungarian (Berkics, 2009), short, 8-item version of the System Justification Scale (Kay and Jost, 2003). Participants rated their agreement with statements, such as “The Hungarian society needs to be radically restructured,” using a scale of 1–9 (1 = *strongly disagree*, 9 = *strongly agree*). The scores was calculated as the average value of the scale items. Cronbach’s α was 0.89.

#### Right-Wing Authoritarianism

*Right-wing authoritarianism* was measured by the Hungarian, 10-item version (Enyedi, 1996) of the *Right-wing Authoritarianism* Scale (Altemeyer, 1981). Participants rated their agreement with statements, such as “It is always better to trust the judgment of the proper authorities in government and religion than to listen to the noisy rabble-rousers in our society, who are trying to create doubt in people’s minds,” on a scale of 1–7 (1 = *strongly disagree*, 7 = *strongly agree*). The score was calculated as the average value of the scale items. Cronbach’s α was 0.89.

#### Social Dominance Orientation

Social dominance orientation was measured with the Hungarian version (Faragó and Kende, 2017) of the SDO7 scale (Ho et al., 2015). Participants rated their agreement with statements, such as “An ideal society requires some groups to be on top and others to be on the bottom,” using a scale of 1–7 (1 = *strongly oppose*, 7 = *strongly favor*). The score was calculated as the average value of the scale items. Cronbach’s α was 0.92.

## Results

### Descriptive Statistics

We first examined all variables to identify outliers. Only one participant, who could not respond to any items on the scientific literacy scale, was removed as an outlier (this also served as a check for random responding).

Means, standard deviations and α values for all scales are presented in Table 2. Some means warrant mention. People were generally dissatisfied with the government (a mean of 3.52 on a scale that consisted of two 11-point items). Real news (regardless of content), although being generally judged as more believable than fake news, were treated with skepticism (a mean 4.37 on a 1–7 scale). Non-political fake news was judged as the least credible, whereas non-political real news was judged as the most believable.

**TABLE 2:** Descriptive statistics and correlations for study variables.

	Variable	*N*	Mean	*SD*	α	1	2	3	4	5	6	7	8	9	10	11	12	13	14	15	16	17	18
1	Anti-government fake news	293	3.40	1.43	0.65	–	0.41**	0.25**	0.13*	–0.04	−0.17**	0.13*	–0.04	–0.10	−0.13*	0.02	−0.44*	0.22*	–0.09	−0.20**	−0.37**	–0.06	–0.03
2	Anti-government real news	293	4.16	1.25	0.40	*0.43* **	–	0.08	0.28**	−0.14*	–0.46	0.02	0.13*	0.12*	0.11	0.15**	−0.33**	–0.02	–0.11	−0.28**	−0.30**	−0.17**	0.03
3	Non-political fake news	294	2.98	1.24	0.37	*0.23* **	*0.09*	–	0.21**	0.42**	0.21**	0.15*	–0.07	−0.20**	−0.18**	–0.10	0.14*	0.29**	0.06	0.26**	0.21**	0.26**	0.06
4	Non-political real news	293	4.37	1.16	0.45	*0.18* *	*0.30* **	*0.29* **	–	0.19**	0.14*	−0.32**	0.40	–0.07	0.02	0.03	–0.06	0.06	0.12*	0.04	–0.08	−0.12*	0.13*
5	Pro-government fake news	293	3.06	1.38	0.71	−*0*.*03*	−*0*.*13*	*0.43* **	*0.18* *	–	0.54**	–0.09	–0.07	−0.3**	−0.18**	−0.20**	0.56**	0.27**	−0.32**	0.54**	0.55**	0.24**	0.02
6	Pro-government real news	293	4.02	1.31	0.34	−0.17*	−*0*.*06*	*0.22* **	*0*.*15*	*0.56* **	–	0.03	0.13*	–0.02	–0.04	0.09	0.58*	0.09	0.26**	0.44*	0.54**	0.12*	0.02
7	Age	278	36.41	15.85	–	–	–	–	–	–	–	–	0.11	0.21**	0.07	0.26**	–0.05	–0.03	−0.15*	–0.01	0.02	0.16**	–0.06
8	Gender	288	1.43	0.52	–	–	–	–	–	–	–	–	–	0.17**	0.24**	0.20**	0.02	0.01	0.18**	0.08	0.08	–0.11	–0.04
9	Political knowledge	293	6.08	2.00	0.57	−*0*.*13*	*0*.*11*	−0.22**	−*0*.*02*	−0.28**	−*0*.*05*	–	–	–	0.33**	0.44**	−0.12*	−0.24**	−0.22**	−0.29**	−0.20**	−0.16**	0.13*
10	Science literacy	293	6.59	1.14	0.23	−*0*.*13*	−*0*.*08*	−0.17*	*0*.*02*	−*0*.*16*	−*0*.*07*	–	–	*0.30* **	–	0.13*	–0.04	–0.12	–0.01	−0.27**	–0.05	−0.15**	0.18**
11	Political interest	295	3.54	1.02	0.87	−*0*.*01*	*0*.*14*	−*0*.*14*	*0*.*11*	−0.18*	*0*.*06*	–	–	*0.39* **	*0*.*07*	–	–0.08	–0.03	–0.09	–0.09	−0.15*	–0.10	0.24**
12	Satisfaction with government	295	3.52	2.55	0.92	−0.46**	−0.34**	*0*.*15*	−*0*.*09*	*0.56* **	*0.58* **	–	–	−*0*.*19*	−*0*.*05*	−*0*.*07*	–	0.05	0.36**	0.64**	0.81**	0.24**	–0.01
13	CMQ	295	4.50	1.19	0.79	*0.23* **	−*0*.*02*	*0.30* **	*0*.*05*	*0.27* **	*0*.*09*	–	–	−0.25**	−*0*.*12*	−*0*.*03*	−*0*.*05*	–	0.20**	0.27**	0.08	0.24**	0.03
14	SDO	284	2.70	1.24	0.92	−*0*.*06*	−*0*.*14*	*0*.*10*	*0*.*06*	*0.33* **	*0.25* **	–	–	−0.24**	−*0*.*04*	−*0*.*10*	*0.36* **	*0.20* *	–	0.50**	0.40**	0.09	–0.02
15	RWA	285	2.19	1.20	0.89	−0.20*	−0.30**	*0.28* **	*0*.*03*	*0.55* **	*0.44* **	–	–	−0.31**	−0.29**	−*0*.*10*	*0.65* **	*0.27* **	*0.49* **	–	0.70**	0.34**	–0.10
16	System justification	286	3.18	1.71	0.89	−0.37**	−0.32**	*0.22* **	−*0*.*09*	–56**	*0.53* **	–	–	−0.22**	−*0*.*07*	−0.17*	*0.81* **	–*08*	*0.40* **	*0.70* **	–	0.26**	–0.02
17	Need for closure	287	3.78	0.81	*0*.*84*	−*0*.*09*	−*0*.*16*	*0.24* **	−*0*.*07*	*0.25* **	*0*.*14*	–	–	−0.19*	−*0*.*14*	−*0*.*13*	*0.25* **	*0.26* **	*0*.*15*	*0.36* **	*0.27* **	–	–19**
18	Openness	292	3.79	0.59	*0*.*66*	−*0*.*02*	*0*.*03*	*0*.*07*	*0*.*12*	*0*.*01*	*0*.*02*	–	–	*0*.*15*	*0.19* *	*0.27* **	−*0*.*01*	*0*.*03*	−*0*.*02*	−*0*.*10*	−*0*.*01*	−0.18*	-

*Correlations in italics are controlled for age and gender.*

**p < 0.001; *p < 0.05.

Pearson correlations coefficients between all variables, with and without controlling for age and gender, are presented in Table 2. Conspiracy mentality correlated with belief in all types of fake news, but not with belief in real news. Satisfaction with government correlated positively with belief in pro-government news and negatively with anti-government news.

### Regressions

We conducted stepwise hierarchical multiple regressions to predict belief in news. These were run separately for each type of news. The independent variables were entered in four steps as follows: (1) age and gender, (2) competencies (objective political knowledge, scientific knowledge and interest in politics), (3) political orientation and (4) those psychological predisposition scales that correlated significantly with the predicted variable (RWA, SDO, SJB, openness, need for closure and/or conspiracy mentality).

#### Anti-government Fake News

Belief in anti-government fake news was correlated with RWA (*r* = −0.20), *p* < 0.001), system justification (*r* = −0.37, *p* < 0.001), and conspiracy mentality (*r* = *0.22*, *p* < 0.001). In step-wise regression, Models 1 and Model 2 were not significant, but Model 3 was significant *R*^2^ = 0.24, *F*(6,270) = 14.18, *p* < 0.001 due to the addition of political orientation Δ*R*^2^ = 0.21, Δ*F*(1,270) = 71.50, *p* < 0.001. The increase in variance explained by the adding the correlated personality dimensions at step 4 was also significant, Δ*R*^2^ = 0.056, Δ*F*(3,263) = 7.01, *p* < 0.001. In Model 4 (*R*^2^ = 0.30 (F(9,263) = 12.31, *p* < 0.001) participants who were older (*B* = 0.02, *t* = 3.00, *p* < 0.01), less satisfied with the government (*B* = −0.21, *t* = −4.14, *p* < 0.001), and scored higher on the conspiracy mentality scale (*B* = 0.28, *t* = 4.27, *p* < 0.001) believed more in anti-government fake news. For all coefficient and model-fit indices see Table 3.

**TABLE 3:** Hierarchical regression results for belief in anti-government fake news.

Variable	*B*	*SE* B	β	*t*	*R* ^2^	*R* ^2^_adjusted_	Δ*R*^2^	AIC	Df
**Model 1**					0.020	0.0129	0.020	981.4525	274
Constant	3.185***	0.301		10.596					
Age	0.012**	0.005	0.133	2.207					
Gender	–0.180	0.166	–0.066	–1.088					
**Model 2**					0.038	0.020	0.018	982.222	271
Constant	3.989***	0.583		6.836					
Age	0.013**	0.006	0.150	2.403					
Gender	–0.087	0.175	–0.032	–0.500					
Objective political knowledge	−0.085*	0.051	–0.115	–1.673					
Scientific literacy	–0.093	0.084	–0.071	–1.111					
Political interest	0.043	0.095	0.031	0.452					
**Model 3**					0.240	0.223	0.208***	919.1493	270
Constant	5.137***	0.537		9.563					
Age	0.013**	0.005	0.141	2.553					
Gender	–0.053	0.156	–0.019	–0.338					
Objective political knowledge	−0.112**	0.045	–0.151	–2.460					
Scientific literacy	–0.083	0.075	–0.063	–1.111					
Political interest	–0.004	0.085	–0.003	–0.051					
Satisfaction with government	−0.262***	0.030	–0.453	–8.456					

**Variable**	***B***	***SE* B**	**β**	***t***	***R* ^2^**	***R* ^2^_adjusted_**	**Δ*R*^2^**	**AIC**	**Df**

**Model 4**					0.296	0.272	0.056***	891.9675	263
Constant	3.808***	0.655		5.815					
Age	0.015***	0.005	0.164	3.002					
Gender	–0.057	0.160	–0.020	–0.354					
Objective political knowledge	−0.082*	0.047	–0.110	–1.757					
Scientific literacy	–0.061	0.076	–0.047	–0.802					
Political interest	–0.047	0.084	–0.033	–0.562					
Satisfaction with government	−0.211***	0.051	–0.366	–4.135					
RWA	–0.016	0.100	–0.013	–0.160					
System justification	–0.107	0.082	–0.125	–1.297					
CMQ	0.284***	0.067	0.236	4.272					

*AIC = Akaike information criterion.*

*p < 0.05; **p < 0.01; ***p < 0.001.

Multicollinearity was not a concern in this or any of the below regressions. VIF scores for all variables were, in all regressions, below 4 and Tolerance was always above 0.169. The assumption of independent errors was always met (Durbin–Watson values = 1.90–2.18).

#### Anti-government Real News

Belief in anti-government real news was correlated with RWA (*r* = −0.28, *p* < 0.001), system justification (*r* = −0.30, *p* < 0.001) and need for closure (*r* = 0.17, *p* < 0.001). Model 1 and Model 2 were not significant, but Model 3 was, Δ*R*^2^ = 0.108, Δ*F*(1,270) = 33.97, *p* < 0.001. Adding the correlated personality dimensions at step 4 did not improve the model. Model 3 indicated that those who were less satisfied with the government (*B* = −0.17, *t* = −5.83, *p* < 0.001), and more interested in politics (*B* = 0.15, *t* = 1.85, *p* < 0.05) believed more in anti-government real news. For all coefficients and model fit indices see Table 4.

**TABLE 4:** Hierarchical regression results for belief in anti-government real news.

Variable	*B*	*SE* B	β	*t*	*R* ^2^	*R* ^2^_adjusted_	Δ*R*^2^	AIC	df
**Model 1**					0.015	0.008	0.015	916.0449	274
Constant	3.749***	0.267		14.035					
Age	0.001	0.005	0.003	0.049					
Gender	0.297**	0.147	0.122	2.016					
**Model 2**					0.037	0.020	0.022*	915.6366	271
Constant	3.045***	0.517		5.886					
Age	–0.003	0.005	–0.036	–0.572					
Gender	0.204	0.155	0.083	1.319					
Objective political knowledge	0.011	0.045	0.017	0.254					
Scientific literacy	0.037	0.074	0.032	0.493					
Political interest	0.178**	0.084	0.144	2.113					
**Model 3**					0.145	0.126	0.108***	884.8132	270
Constant	3.789***	0.505		7.505					
Age	–0.003	0.005	–0.041	–0.706					
Gender	0.227	0.146	0.093	1.550					
Objective political knowledge	–0.006	0.043	–0.009	–0.135					
Scientific literacy	0.043	0.070	0.037	0.616					
Political interest	0.148*	0.080	0.119	1.849					
Satisfaction with government	−0.170***	0.029	–0.331	–5.828					

**Variable**	***B***	***SE* B**	**β**	***t***	***R* ^2^**	***R* ^2^_adjusted_**	**Δ*R*^2^**	**AIC**	**df**

**Model 4**					0.161	0.132	0.016	873.9096	262
Constant	4.665***	0.682		6.844					
Age	–0.001	0.005	–0.009	–0.145					
Gender	0.269*	0.156	0.108	1.723					
Objective political knowledge	–0.027	0.045	–0.041	–0.603					
Scientific literacy	0.023	0.074	0.020	0.313					
Political interest	0.121	0.082	0.097	1.483					
Satisfaction with government	−0.126**	0.050	–0.245	–2.529					
RWA	–0.022	0.097	–0.020	–0.228					
System justification	–0.060	0.080	–0.080	–0.758					
Need for closure	–0.153	0.099	–0.097	–1.545					

*AIC = Akaike information criterion.*

*p < 0.05; **p < 0.01; ***p < 0.001.

#### Non-political Real News

Belief in non-political real news was correlated with SDO (*r* = 0.12, *p* < 0.05) and openness (*r* = 0.13, *p* < 0.05). Regression Model 1 was significant, *R*^2^ = 0.11, *F*(2,274) = 17.48, *p* < 0.001, and additional steps could not contribute to it. Model 1 indicated that younger (*B* = −0.02, *t* = −5.824, *p* < 0.001) and male (*B* = 0.22, *t* = 1.17, *p* < 0.05), participants believed more in non-political real news. For all coefficients and model-fit indices see Table 5.

**TABLE 5:** Hierarchical regression results for belief in non-political real news.

Variable	*B*	*SE* B	β	*t*	*R* ^2^	*R* ^2^_adjusted_	Δ*R*^2^	AIC	df
**Model 1**					0.113	0.107	0.113***	843.7849	274
Constant	4.951	0.234		21.123					
Age	−0.024***	0.004	–0.333	–5.824					
Gender	0.216*	0.129	0.096	1.671					
**Model 2**					0.126	0.110	0.013	845.7849	271
Constant	4.687***	0.456		10.276					
Age	−0.025***	0.004	–0.349	–5.888					
Gender	0.181	0.136	0.080	1.329					
Objective political knowledge	–0.048	0.040	–0.079	–1.203					
Scientific literacy	0.023	0.065	0.021	0.348					
Political interest	0.140	0.074	0.122	1.882					
**Model 3**					0.131	0.011	0.005	846.1861	270
Constant	4.836***	0.471		10.268					
Age	−0.260***	0.004	–0.350	–5.915					
Gender	0.186	0.136	0.082	0.174					
Objective political knowledge	–0.051	0.040	–0.084	–1.288					
Scientific literacy	0.024	0.070	0.022	0.369					
Political interest	0.133	0.074	0.170	1.798					
Satisfaction with government	–0.034	0.027	–0.071	–1.250					

**Variable**	***B***	***SE* B**	**β**	***t***	***R* ^2^**	***R* ^2^_adjusted_**	**Δ*R*^2^**	**AIC**	**df**

**Model 4**					0.143	0.116	0.012	831.2888	262
Constant	4.228***	0.604		7.001					
Age	−0.025***	0.004	–0.336	–5.510					
Gender	0.192	0.147	0.083	1.308					
Objective political knowledge	–0.039	0.041	–0.064	–0.947					
Scientific literacy	0.008	0.067	0.007	0.115					
Political interest	0.109	0.078	0.095	1.402					
Political orientation	–0.047	0.029	–0.099	–1.589					
SDO	0.064	0.062	0.067	1.024					
Openness	0.150	0.121	0.075	1.233					

*AIC = Akaike information criterion.*

*p < 0.05; ***p < 0.001.

#### Non-political Fake News

Belief in non-political fake news was correlated with RWA (*r* = *0.26*, *p* < 0.001), system justification (*r* = *0.21*, *p* < 0.001), need for closure (*r* = 0.26, *p* < 0.001) and CMQ (*r* = 0.29, *p* < 0.001). Model 1 was significant (*p* < 0.05), but Steps 2, 3 [Δ*R*^2^ = 0.06, ΔF(1,271) = 9.13, *p* < 0.01] and 4 [Δ*R*^2^ = 0.07, Δ*F*(4,261) = 6.48, *p* < 0.001] all improved the model [Model 2, *R*^2^ = 0.09, *F*(5,272) = 5.46, *p* < 0.001; Model 3, *R*^2^ = 0.12, *F*(6,271) = 6.21, *p* < 0.001, Model 4, *R*^2^ = 0.20, *F*(10,261) = 6.30, *p* < 0.001]. Model 4 shows that participants who were older (*B* = 0.01, *t* = 2.67, *p* < 0.01) had higher need for closure (*B* = 0.20, *t* = 3.42, *p* < 0.05) and scored higher on conspiracy mentality (*B* = 0.21, *t* = 3.42, *p* < 0.001) believed more in non-political fake news. For all coefficients and model-fit indices, see Table 6.

**TABLE 6:** Hierarchical regression results for belief in non-political fake news.

Variable	*B*	*SE* B	β	*t*	*R* ^2^	*R* ^2^_adjusted_	Δ*R*^2^	AIC	df
**Model 1**					0.029	0.022	0.029*	900.7383	275
Constant	2.769***	0.258		10.730					
Age	0.012***	0.005	0.159	2.666					
Gender	–0.195	0.142	–0.082	–1.374					
**Model 2**					0.091	0.074	0.062***	888.3839	272
Constant	4.193***	0.490		8.562					
Age	0.017***	0.004	0.215	3.563					
Gender	–0.020	0.146	–0.008	–0.137					
Objective political knowledge	−0.107*	0.043	–0.167	–2.517					
Scientific literacy	–0.120	0.070	–0.106	–1.708					
Political interest	–0.105	0.079	–0.087	–1.325					
**Model 3**					0.121	0.101	0.059**	881.1735	271
Constant	3.813	0.499		7.647					
Age	0.017***	0.005	0.218	3.671					
Gender	–0.029	0.144	–0.012	–0.205					
Objective political knowledge	−0.098*	0.042	–0.152	–2.321					
Scientific literacy	–0.123	0.069	–0.109	–1.784					
Political interest	–0.091	0.078	–0.075	–1.164					
Satisfaction with government	0.087**	0.029	0.173	3.021					

**Variable**	***B***	***SE* B**	**β**	***t***	***R* ^2^**	***R* ^2^_adjusted_**	**Δ*R*^2^**	**AIC**	**df**

**Model 4**					0.194	0.163	0.073***	848.2895	262
Constant	1.542*	0.680		2.267					
Age	0.012**	0.005	0.160	2.666					
Gender	–0.120	0.147	–0.050	–0.806					
Objective political knowledge	–0.029	0.043	–0.045	–0.669					
Scientific literacy	–0.080	0.070	–0.071	–1.063					
Political interest	–0.081	0.070	–0.068	–1.063					
Political orientation	0.001	0.047	0.001	0.007					
RWA	0.041	0.093	0.039	0.443					
System justification	0.102	0.076	0.139	1.351					
Need for closure	0.204*	0.096	0.133	3.421					
CMQ	0.214***	0.062	0.206	3.421					

*AIC = Akaike information criterion.*

*p < 0.05; **p < 0.01; ***p < 0.001.

#### Pro-government Fake News

Belief in pro-government fake news was correlated with RWA (*r* = 0.54, *p* < 0.001), system justification (*r* = 0.55, *p* < 0.001), conspiracy mentality (*r* = *0.27*, *p* < 0.001), need for closure (*r* = 0.24, *p* < 0.001) and SDO (*r* = 0.32, *p* < 0.001). Model 1 was not significant, but Model 2 was [*R*^2^ = 0.11, *F*(5,271) = 6.90, *p* < 0.001], and adding political orientation in Step 3 [Δ*R*^2^ = 0.25, Δ*F*(1,270) = 104.70, *p* < 0.001] further increased the amount of explained variance [Model 3, *R*^2^ = 0.36, *F*(6,270) = 25.40, *p* < 0.001], as well as did adding the correlated personality dimensions in Step 4 [Δ*R*^2^ = 0.05, Δ*F*(5,259) = 4.60, *p* < 0.001; Model 4, *R*^2^ = 0.42, *F*(11,259) = 16.71, *p* < 0.001]. Participants who were more satisfied with the government (*B* = 0.16; *t* = 3.39, *p* < 0.001), scored higher in on conspiracy mentality (*B* = 0.19; *t* = 3.12, *p* < 0.001) and had higher system justification (*B* = 0.17, *t* = 2.28, *p* < 0.01) beliefs rated pro-government fake news as more accurate. For all coefficients and model-fit indices, see Table 7.

**TABLE 7:** Hierarchical regression results for belief pro-government fake news.

Variable	*B*	*SE* B	β	*t*	*R* ^2^	*R* ^2^_adjusted_	Δ*R*^2^	AIC	df
**Model 1**					0.011	0.005	0.011	956.172	274
Constant	3.518***	0.287		12.253					
Age	–0.007	0.005	–0.081	–1.352					
Gender	–0.167	0.158	–0.064	–1.054					
**Model 2**					0.112	0.101	0.101***	932.3040	271
Constant	5.198***	0.553		9.750					
Age	–0.001	0.005	–0.001	–0.070					
Gender	0.050	0.160	0.019	0.312					
Objective political knowledge	−0.162***	0.046	–0.229	–3.475					
Scientific literacy	–0.88	0.076	–0.071	–1.155					
Political interest	−0.182**	0.087	–0.137	–2.102					
**Model 3**					0.361	0.347	0.249***	843.5304	270
Constant	3.986***	0.469		8.505					
Age	0.001	0.004	0.004	0.093					
Gender	0.013	0.136	0.005	0.097					
Objective political knowledge	−0.133***	0.040	–0.189	–3.367					
Scientific literacy	–0.098	0.065	–0.080	–1.523					
Political interest	−0.133*	0.074	–0.010	–1.179					
Satisfaction with government	0.276***	0.027	0.502	10.232					

**Variable**	***B***	***SE* B**	**β**	***t***	***R* ^2^**	***R* ^2^_adjusted_**	**Δ*R*^2^**	**AIC**	**df**

**Model 4**					0.415	0.390	0.054***	815.2916	259
Constant	2.367***	0.647		3.657					
Age	–0.001	0.004	–0.010	–0.197					
Gender	–0.111	0.143	–0.042	–0.780					
Objective political knowledge	−0.076*	0.042	–0.106	–1.840					
Scientific literacy	–0.064	0.068	–0.051	–0.944					
Political interest	–0.121	0.074	–0.090	–1.637					
Political orientation	0.155***	0.046	0.281	3.394					
RWA	0.079	0.093	0.067	0.400					
System justification	0.166**	0.073	0.203	2.278					
Conspiracy mentality	0.186***	0.060	0.162	3.119					
Need for closure	0.002	0.091	0.001	0.022					
Social dominance orientation	0.038	0.065	0.034	0.582					

*AIC = Akaike information criterion.*

*p < 0.05; **p < 0.01; ***p < 0.001.

#### Pro-government Real News

Belief in pro-government real news was correlated with RWA (*r* = 0.44, *p* < 0.001), system justification (*r* = 0.54, *p* < 0.001), need for closure (*r* = 0.12, *p* < 0.001) and SDO (*r* = 0.26, *p* < 0.001). Model 1 and Model 2 were not significant, but Model 3 [*R*^2^ = 0.33, *F*(6,270) = 21.66, *p* < 0.001] was and adding the correlated personality dimensions in step 4 further improved the model [Δ*R*^2^ = 0.03, Δ*F*(4,260) = 2.25, *p* < 0.05]. Participants who had higher scores in political interest (*B* = 0.16, *t* = 2.22, *p* < 0.01), were more satisfied with the government (*B* = 0.187, *t* = 4.09, *p* < 0.001), were male (*B* = 0.220, *t* = 0.09, *p* < 0.05) and had higher SJB (*B* = 0.18, *t* = 2.50, *p* < 0.01) believed more in pro-government real news. For all coefficients and model-fit indices, see Table 8.

**TABLE 8:** Hierarchical regression results for belief in pro-government real news.

Variable	*B*	*SE* B	β	*t*	*R* ^2^	*R* ^2^_adjusted_	Δ*R*^2^	AIC	df
**Model 1**					0.018	0.011	0.018*	927.9624	274
Constant	3.488***	0.272		12.781					
Age	0.001	0.005	0.016	0.274					
Gender	0.329**	0.150	0.132	2.187					
**Model 2**					0.024	0.006	0.006	932.4005	271
Constant	3.722***	0.533		6.980					
Age	0.001	0.005	0.011	0.177					
Gender	0.350**	0.159	0.140	2.197					
Objective political knowledge	–0.028	0.046	–0.042	–0.612					
Scientific literacy	–0.053	0.076	–0.045	–0.695					
Political interest	0.079	0.087	0.062	0.906					
**Model 3**					0.325	0.310	0.301***	832.1877	270
Constant	2.448***	0.460		5.331					
Age	0.002	0.004	0.020	0.400					
Gender	0.312**	0.133	0.125	2.347					
Objective political knowledge	0.001	0.389	0.002	0.028					
Scientific literacy	–0.064	0.064	–0.054	–1.011					
Political interest	0.131*	0.072	0.104	1.809					
Satisfaction with government	0.290***	0.026	0.554	1.809					

**Variable**	***B***	***SE* B**	**β**	***t***	***R* ^2^**	***R* ^2^_adjusted_**	**Δ*R*^2^**	**AIC**	**df**

**Model 4**					0.351	0.327	0.026*	816.2556	260
ß	2.121***	0.618		3.433					
Age	0.001	0.004	0.008	0.139					
Gender	0.220*	0.143	0.086	1.533					
Objective political knowledge	0.029	0.041	0.042	0.072					
Scientific literacy	–0.066	0.068	–0.055	–0.965					
Political interest	0.164**	0.074	0.129	2.218					
Political orientation	0.187***	0.046	0.355	4.085					
RWA	0.020	0.093	0.018	0.217					
System justification	0.181**	0.072	0.233	2.501					
Need for closure	–0.037	0.090	–0.024	–0.430					
Social dominance orientation	0.040	0.065	0.038	0.630					

*AIC = Akaike information criterion.*

*p < 0.05; **p < 0.01; ***p < 0.001.

## Discussion

The aim of this study was to examine social psychological factors associated with belief in fake and real news, while controlling for demographics. We examined this in the politically polarized context of Hungary, where the proliferation of disinformation is high.

We found that the only psychological predisposition that was consistently associated with belief in any type of fake news (political and non-political) was conspiracy mentality. Regarding other underlying determinants of tendencies to believe fake and real news, we found that clear support for the motivational reasoning explanation as political orientation consistently predicted belief in both fake and real political news when their contents aligned with one’s political identity. The belief in pro-government news was also associated with higher SJB among pro-government supporters. Those interested in politics showed better capacity to distinguish real political news from the fake ones. Demographics did not play an explanatory role.

Our results corroborated that conspiracy mentality was associated with believing all types of fake news including political (pro- and anti-government) as well as non-political. Despite lacking shared narrative or sentiment (e.g., the news items were not all threatening), fake news were more plausible to people with higher levels of conspiracy mentality. Why exactly this was the case should be an interesting avenue for future research. The fake news that we presented to participants are likely to have differed from other news on various covarying dimensions (e.g., falsifiability, person-centeredness, use of statistical data, information source), and in retrospect it is impossible to determine which of these dimensions is relevant when determining why these news were particularly plausible to those with a proneness to believe in conspiracies. This needs to be investigated much more systematically and vigorously.

Importantly, the belief in fake news was not confined to the belief in the conservative right-wing government’s narrative. This is in line with previous research suggesting that conspiracy mentality is not confined to a certain political ideology but can be more common on the extremes of the political spectrum (e.g., van Prooijen et al., 2015). Furthermore, conspiracy theories are associated with political polarization, rather than a specific ideology (Sutton and Douglas, 2020). As Hungary can be characterized by high levels of political polarization, belief in conspiracy theories may be particularly high. Moreover, it is important that whether the news was anti- or pro-government did not matter. This could be because in Hungary people all over the ideological field can be found in both camps. In a context “cleansed” of potentially confounding ideological factors, it thus seems that conspiracy mentality is in its own right an important determinant of fake news susceptibility.

In the case of political fake and real news, participants exhibited bias according to their political preferences—that is, they believed the political news that was congruent with their political outlook toward the government. Being anti-government predicted believing both fake and real anti-government news, whereas being pro-government predicted believing both fake and real pro-government news. These results replicated some previous findings (e.g. Pennycook and Rand, 2018; Calvillo et al., 2021) and are in contrast to some previous findings that have suggested that right-wing or conservative-leaning individuals may be more prone to believe or share disinformation (e.g., Allcott and Gentzkow, 2017; Jost, 2017).

It is also worthwhile to note that SJB were associated with belief in pro-government fake and real news (but no other types of news). System justification can be described as a basic need to validate the existing social arrangements and systems (Jost and Hunyady, 2005)—it seems natural that those who are strongly motivated to believe in the system in which they live would endorse information that supports that system, regardless of the accuracy of this information. This is consistent with the notion that the motivation to believe that the system is just can lead to the perceived legitimacy of authorities and institutions (Jost and Hunyady, 2005).

Belief in conspiracies, suggested to stem from epistemic, existential, and social motives (Douglas et al., 2017), has been argued to be a generalized political attitude (e.g., Bruder et al., 2013) as well as a group-based phenomenon (e.g., Jasinskaja-Lahti and Jetten, 2019). Conspiracy theories have also been associated with motivated reasoning, especially in terms of partisanship and political ideology (e.g., Uscinski and Parent, 2014; Douglas et al., 2019). In this view, people not only filter information and events through their political identities, but also assess the role of conspiracies through a political lens (Brotherton et al., 2013).

It seems that the conspiracy mentality, could, across various context, reflect some deeper predisposition to believe in fake news that is independent of specific political views or other political attitudes. Our results appear to be highly consistent with such a view, as we have found no other attitudes or traits (RWA, SDO, SJB, openness and need for cognitive closure) that would have consistently been associated with fake news belief. It is possible that belief in conspiracies and belief in disinformation work in similar ways and can satisfy epistemic motivations to build a stable and consistent understanding of the world. The Hungarian government’s populist communication has largely been focusing on the construction of powerful common enemies (Hegedüs, 2019), who—as in conspiratorial narratives—are secretly plotting against the nation and its values. As such, supporters of the government can be affected by these narratives. Meanwhile, on the anti-government side, a conspiracy mentality could, in the absence of sociopolitical control, be more related to existential motivations (e.g., to help feel more secure in the face of oppression). Thus, such a society could provide a fertile ground for a conspiracy mentality and susceptibility to fake news. What epistemic, existential, and social motives are actually served by conspiracy theories putting forth different types of narratives in a context such as Hungary should be an interesting question for future research.

Furthermore, competencies such as political and scientific knowledge did not protect against gullibility, highlighting the importance of psychological predispositions and orientations over knowledge. Interestingly, however, those who claimed to be more interested in politics, were more accurate in discerning real from fake news when the news were political (i.e., anti- or pro-government) despite not knowing more about politics than those who were less interested. This pattern is difficult to interpret, and our results have to be more generally interpreted as suggesting that interventions focusing on knowledge may not be effective in countering disinformation. On the other hand psychological predispositions, such as conspiracy mentality, are rather constant, suggesting they are also a poor target for interventions. It might be that those motivated to believe in conspiracies will continue doing so and targeting the individual in the fight against disinformation may not be very fruitful. Instead, the focus could be targeted on increasing the interest of people toward politics and social issues, which could counteract the stable negative role of conspiracy mentality.

### Limitations and Future Directions

Perhaps the most obvious limitation of our research is our reliance on a convenience sample—participants were volunteers recruited through social media, and the sample was not representative of the population of Hungary. However, our focus was on fake news, and these tend to be disseminated through social media, which means that we are likely to have reached exactly those Hungarians who also tend to encounter fake news. Regarding self-selection, one could argue that participants to survey research are always volunteers, and there is no reason to think that our participants are particularly self-selected. For instance, as compared to the nationally representative 2018 European Social Survey (ESS), our sample was similarly unsatisfied with the government [our sample’s average was *M* = 3.80 (*SD* = 2.55), the ESS average was *M* = 3.52, SD = 2.55]. Regarding the participants, the failure rate was rather high which might be due to the poor level of attention that participants exhibit during on online questionnaire which is not being administered in controlled circumstances.

To measure conspiracy mentality, we relied on the Conspiracy Mentality Questionnaire, an instrument developed to measure generic conspiracy ideation (Bruder et al., 2013). Although the instrument has been validated across cultures, some of the items may be more open to interpretation in an authoritarian context, for instance “*politicians may not always tell the true motives for their decisions.*” Such statements could appear especially plausible among those opposing the current government, adding variance that may reflect subjectively perceived democracy and/or discontent with the current power relations more than conspiracy mentality *per se*.

It is worth mentioning is that all news items were presented without a source. This allowed us to exclude the potential effects of the source on participants. However, the source of the news could be an important factor affecting how people process and judge news. Future work should investigate the role of source in the processing of news, both fake and real. Furthermore, even though the news selection followed certain criteria, it might still have been subjective to a certain degree. The Cronbach’s α for the news scale was on the lower end, which can be because they only contained only three items per scale. However, this may not be a serious limitation, as Cronbach’s α has been convincingly shown to have several issues as a measure of reliability (McCrae et al., 2011; McNeish, 2018). We also included the Spearman-Brown index, suggested to be preferable for very short scales (McCrae et al., 2011), as an alternative measure of reliability. Regarding the news stories, as already alluded to above, future research could explore what makes fake news different from real news in the eyes of people, in terms of complexity, elicited emotions or interest. Such studies could more effectively capture the persuasion mechanisms that fake news utilize.

## Conclusion

In conclusion, our study highlighted the importance of conspiracy mentality in judgments of political and non-political fake news belief. Other psychological predispositions (SDO, RWA, SJB, openness, need for closure) were not associated with fake news belief. Moreover, competencies such as political and scientific knowledge and political interest did not guard against gullibility toward fake news.

Future research should focus on the relationship between psychological predispositions and the wider socio-political context. In order to understand why people believe and share false information, we need to know more about how the individual interacts with their online and wider environment in which they consume (false) information. This way, we can think of both macro and micro level interventions and policies that can hinder the spread of false information.

Our results also draw attention to the need for studying existing concepts and phenomena in other than Western contexts. Most studies on this topic have been done in a Western context, with theories developed in a similar milieu. By studying extreme contexts, such as Hungary, in which the proliferation of disinformation is high and the media landscape is almost completely controlled by a state that actively pushes disinformation, we can gain valuable insight into the characteristics of individuals who are most resistant to disinformation, even in such extreme contexts, and potentially improve people’s ability to recognize false information online.

## Data Availability Statement

The datasets presented in this study can be found in online repositories. The names of the repository/repositories and accession number(s) can be found below: https://osf.io/zqdm7/.

## Ethics Statement

The studies involving human participants were reviewed and approved by the Eötvös Loránd University Ethical Committee. The patients/participants provided their written informed consent to participate in this study.

## Author Contributions

ZS contributed to all aspects of work in this article, produced the initial drafts, and conducted the main statistical analysis. J-EL contributed to the conceptualizing and developing the study. J-EL and IJ-L have made substantial contributions to the theoretical introduction and the discussion, as well as to the analytical choices and to revising the article critically. All authors contributed to the article and approved the submitted version.

## Conflict of Interest

The authors declare that the research was conducted in the absence of any commercial or financial relationships that could be construed as a potential conflict of interest.

## Publisher’s Note

All claims expressed in this article are solely those of the authors and do not necessarily represent those of their affiliated organizations, or those of the publisher, the editors and the reviewers. Any product that may be evaluated in this article, or claim that may be made by its manufacturer, is not guaranteed or endorsed by the publisher.
