# Supplementary material for: Social Psychological Predictors of Belief in Fake News in the Run-Up to the 2019 Hungarian Elections: The Importance of Conspiracy Mentality Supports the Notion of Ideological Symmetry in Fake News Belief
Source: Front Psychol. 2021 Dec 24;12:790848. doi: 10.3389/fpsyg.2021.790848 (PMC8740309; doi:10.3389/fpsyg.2021.790848)
Supplement: Supplementary file 1 [file Data_Sheet_1.pdf]

1 News items used in the study

Note: In the study the news items were presented with headline and byline (see below) and a picture, captured as a screenshot.

1.1 Anti-government narrative, fake

A whole floor is reserved for Viktor Orban, at a secret private clinic in Graz

Our source was not willing to tell us anything about the illness of the prime minister, but they said, that the most modern equipment and and neurologists who studied at the best places are at the disposal of the important guest.

"The country is controlled by the criminal organisation of Sandor Pinter, interior minister"

Said Tamas Portik at court

An audio has been leaked, where Fidesz admits to the election freud of 2018

Szilard Nemeth accidentaly admits

1.2 Anti-government narrative, real

Zoltan Kovacs earned a doctorate from CEU by self-plagiarism

Zoltan Kovacs, government spokesperson earned two history doctorates after another, one from the University of Debrecen, and one from CEU in the early 2000s

Median: In 3 months Fidesz-KDNP lost half-million supporters

According to January polls, in 3 months 500,000 supporters left Fidesz

Here is the letter of Orban, asking for George Soros' help

Most people know, that Viktor Orban studied at Oxford before the end of communism in Hungary. 30 years passed, and many things changed in the mind of the prime minister.

1.3 Pro-government narrative, fake

There is a huge amount of money in the secret bank account of Ferenc Gyurcsany

The president of the Democratic Coalition has vast amount of wealth in an Austrian bank, which does not appear in his taxes

Simicska bought Vona with an Audi, worth 20 million, with a startling license plate

Lajos Simicska surprised the president of Jobbik with an expensive gift

Peter Juhasz regularly abused drugs in front of his children

As we earlier reported, shocking details came to light from the files of the court case of Peter Juhasz: the partner of the president of Egyutt said, that he gave her tranquilizers , after he knelt on her and hit her

1.4 Pro-government narrative, real

GDP grew with 4.8% last year

Hungary's gross domestic product grew with 4.8% in last year's last quarter, when accounting for seasonal and calendar effects. According to raw data, it grew by 5% compared to the same period of the year before – reported the KSH Thursday morning.

Violent crime grew in Germany, caused by migrants

Violent crime grew in Germany with 10% in 2015 and 2016. 90% of the growth is connected to young (aged between 14 and 30) male refugees/migrants.

Heil Sneider: they posed with a nazi salute at the wedding of the president of Jobbik

While left-wing analysts and politicians are discussing whether their voters are willing to vote for Jobbik, which has abandoned its past ideology and started to move towards being a people's party,

1.5 Nonpolitical, fake

Herb man from Bükk: everything is healable!

To prevent cancer: soda bicarbonate, for childless: celery and quail egg yolk!

They found a giant snake, just like in nightmares

10 meter long, 400 kg, 1 meter diameter nightmare

298 healthy birds died when testing the 5G mobile technology!

It has been discussed whether the new mobile technology is dangerous or not. 5G has not been introduced yet, but what happened in The Hague, is gruesome

1.6 Nonpolitical, real

Archaeologists found an untouched, thousand-year old Maya cave in Mexico

In the ruins of Chichén Itzá they also found objects which has been sacrificed to the god of rain. Based on this, researchers are examining the consequences of draught, as a reason for the fall of the Maya

Pedestrians die because of healthy lifestyle, mobile phones and big cars

Since 1990, last year was the highest in pedestrian deaths caused by accidents

Sinister report: in 11 years, summers might be ice-free on the North Pole

The ocean around the North Pole might become ice-free much earlier, maybe by the summer of 2030 according to a news study
